# Inpatient care for stiff person syndrome in the United States: a nationwide readmission study

**DOI:** 10.1186/s40734-018-0071-9

**Published:** 2018-08-06

**Authors:** James A. G. Crispo, Dylan P. Thibault, Yannick Fortin, Allison W. Willis

**Affiliations:** 1 Department of Neurology, University of Pennsylvania Perelman School of Medicine, Blockley Hall, 423 Guardian Drive, Office 829, Philadelphia, PA 19104 USA; 2 Department of Biostatistics, Epidemiology and Informatics, University of Pennsylvania Perelman School of Medicine, Blockley Hall, 423 Guardian Drive, Office 811, Philadelphia, PA 19104 USA; 3 Department of Neurology Translational Center of Excellence for Neuroepidemiology and Neurological Outcomes Research, University of Pennsylvania Perelman School of Medicine, Philadelphia, PA USA; 4 McLaughlin Centre for Population Health Risk Assessment & Interdisciplinary School of Health Science, Faculty of Health Sciences, University of Ottawa, 850 Peter Morand Crescent, Room 119, Ottawa, ON K1G 3Z7 Canada; 5 Center for Clinical Epidemiology and Biostatistics, University of Pennsylvania Perelman School of Medicine, Blockley Hall, 423 Guardian Drive, Office, Philadelphia, PA 19104 USA

**Keywords:** Stiff person syndrome, Inpatients, Readmission, United States, Rare disorder

## Abstract

**Background:**

Stiff person syndrome (SPS) is a progressive neurological disorder characterized by axial muscle rigidity and involuntary spasms. Autoimmune and neoplastic diseases are associated with SPS. Our study objectives were to describe inpatient care for SPS in the United States and characterize 30-day readmissions.

**Methods:**

We queried the 2014 Nationwide Readmission Database for hospitalizations where a diagnosis of SPS was recorded. For readmission analyses, we excluded encounters with missing length of stay, hospitalization deaths, and out-of-state and December discharges. National estimates of index hospitalizations and 30-day readmissions were computed using survey weighting methods. Unconditional logistic regression was used to examine associations between demographic, clinical, and hospital characteristics and readmission.

**Results:**

There were 836 patients with a recorded diagnosis of SPS during a 2014 hospitalization. After exclusions, 703 patients remained, 9.4% of which were readmitted within 30 days. Frequent reasons for index hospitalization were SPS (27.8%) and diabetes with complications (5.1%). Similarly, readmissions were predominantly for diabetes complications (24.2%) and SPS. Most readmissions attributed to diabetes complications (87.5%) were to different hospitals. Female sex (OR, 3.29; CI: 1.22–8.87) and routine discharge (OR, 0.26; CI: 0.10–0.64) were associated with readmission, while routine discharge (OR, 0.18; CI: 0.04–0.89) and care at for-profit hospitals (OR, 10.87; CI: 2.03–58.25) were associated with readmission to a different hospital.

**Conclusions:**

Readmissions in SPS may result from disease complications or comorbid conditions. Readmissions to different hospitals may reflect specialty care, gaps in discharge planning, or medical emergencies. Studies are required to determine if readmissions in SPS are preventable.

## Background

First described by Moersch and Woltman [1], stiff person syndrome (SPS) (formerly referred to as stiff man syndrome) is a rare and progressive autoimmune disorder that is characterized by rigidity and stiffness of axial and lower limb muscles, as well as painful involuntary spasms that may lead to patient disability [1]. Although the pathophysiology of classic SPS remains to be fully elucidated, there are known immunological markers for SPS and an autoimmune link between diabetes mellitus (type 1) and SPS involving the neuroendocrine autoantibody specific for glutamic acid decarboxylase (65 kD isoform; GAD65) has been demonstrated [2–4]. While GAD65 positivity is common in the general population, individuals with SPS can have markedly elevated levels of GAD65 and often respond positively to immunotherapy [5, 6]. Individuals with SPS may also exhibit elevated titers for secondary SPS markers, including antibodies against gamma-aminobutyric acid A receptor protein and the glycine-alpha 1 receptor. Testing for these other antibodies is recommended, particularly in individuals with low GAD65 and symptoms of SPS [2, 5, 7]. Paraneoplastic SPS, a non-classic form of the disease reported to occur in approximately 5% of cases [4], is most common among patients with breast cancer, followed by colon cancer and lung cancer.

During a 25-year period (1984–2008), the Mayo Clinic reported caring for approximately 4 distinct individuals with classic SPS annually, two thirds of whom were women [6]. It has also been reported that 119 unique cases of SPS were identified among the entire United Kingdom population between 2000 and 2005, which coincides with prior suggestions that the prevalence of SPS is approximately 1–2 cases per million individuals [8]. To date, onset, progression, and treatment of SPS is primarily described by case reports. There is therefore a paucity of population-based data on SPS hospitalizations and subsequent readmissions.

To increase knowledge of inpatient care for SPS, we used the 2014 Healthcare Cost and Utilization Project (HCUP) Nationwide Readmission Database to identify individuals hospitalized with SPS and assess their utilization of health services. Our primary objectives were to characterize inpatients with SPS and quantify their 30-day readmission rates. Awareness of primary causes of readmission and factors associated with acute readmission may contribute to improved discharged planning, outpatient follow-up, and health outcomes. Our secondary objectives were therefore to identify primary reasons for readmission within 30 days of hospital discharge and examine whether certain demographic, clinical, and care setting characteristics were associated with inpatient readmission.

## Methods

### Ethics statement

This study was exempt from research ethics board review since it involved secondary analyses of de-identified health claims data and complied with conditions outlined in the United States Agency for Healthcare Research and Quality’s (AHRQ) Healthcare Cost and Utilization Project (HCUP) Data Use Agreement. The HCUP Data Use Agreement prohibits reporting of cell sizes ≤10; therefore, results were suppressed as appropriate.

### Data source

This study was conducted using administrative health data from the 2014 Nationwide Readmissions Database (NRD). Sponsored by the AHRQ, the NRD is a family of databases developed as part of the HCUP to support national readmission analyses. Available data include health service utilization information for all health insurance payer categories in the United States, including the uninsured. The NRD contains detailed demographic (such as age, sex, and health insurance status), clinical (such as diagnoses, procedures, and length of stay), and hospital data (such as bed size, location, and teaching status) that may be weighted to generate nationally representative estimates of hospitalizations and subsequent readmissions for individuals of all ages. Inpatients in NRD datasets may be tracked longitudinally within but not across calendar years or states.

### Study population

The 2014 NRD was queried to identify index SPS encounters, which were defined as hospitalizations where an International Classification of Diseases, Ninth Revision (ICD-9) diagnosis code for SPS (333.91) was recorded as a primary or secondary diagnosis. Encounters where length of stay was undocumented or where the patient died were then excluded. It is not possible to track individuals in the NRD across state borders; therefore, index SPS encounters occurring outside of the patient’s home state were excluded. To ensure that all hospital readmissions within 30 days of the index encounter could be identified, index SPS encounters discharged in the month of December were also excluded. In instances where patients had multiple eligible SPS index encounters, a single index encounter was randomly selected for our analyses.

### Inpatient demographics, comorbidities, and hospital characteristics

Demographic and clinical data extracted from index SPS encounters included patient age, sex, health insurance payer category, patient median household income, length of stay, and discharge disposition. Comorbidities recorded during the index SPS hospitalizations were ascertained using the ICD-9 Elixhauser comorbidity measures, which were previously validated in administrative claim and electronic health record data [9, 10]. A pooled morbidity score was then computed by summing the number of prevalent comorbidities measures recorded during the index hospitalization. Hospital characterisitcs examined during index encounters included hospital size, control/ownership, and teaching status.

### Readmissions

Eligible readmissions included all-cause elective or non-elective readmissions within 30 days of the index encounter discharge date. For patients readmitted to hospital within 30 days, time to readmission was calculated as the number of days separating the discharge date of the index encounter and the earliest hospital readmission date. Subsequent readmissions within the 30-day follow-up period were ignored.

### Statistical analyses

Nationally representative estimates of SPS index event characteristics (demographic, clinical, and hospital) and 30-day readmission (rates, reasons, and time to readmission) were calculated using survey weighting methods and reported using descriptive statistics. Primary reasons for index hospitalization and 30-day readmission were grouped using the HCUP single-level Clinical Classifications Software [11], a classification scheme that enables individual ICD-9 codes to be categorized according to clinical similarities. The 10 most common reasons for index hospitalization and readmission were reported in order of decreasing prevalence, with readmissions further categorized according to care setting (readmission to the same or to a different hospital). To examine associations between demographic, clinical, and hospital characteristics and readmission within 30 days of the index SPS encounter discharge, we fitted weighted unconditional logistic regression models to estimate the crude odds of all-cause readmission to any hospital and to a different hospital. Due to the exploratory nature of this study, adjustments were not made for multiple comparisons. Statistical analyses were performed with SAS v9.4 (SAS Institute Inc., Cary, NC, USA).

## Results

### Cohort and hospital characteristics

There were 836 distinct individuals with a recorded diagnosis of SPS during a 2014 hospitalization. After applying study exclusion criteria, there were 703 hospitalized individuals with SPS who were discharged between January 1, 2014 and November 30, 2014 (Table 1). Our data were consistent with previously published descriptive epidemiological data on SPS. The majority of admitted patients were 40–59 years of age (46.1%) and few patients (15.8%) were younger than 40 years of age at admission. Mean patient age was 53.7 ± 0.9 (standard error) years. Nearly two thirds of admitted patients were female (63.9%) and the majority of patients were covered by publicly funded health insurance plans (63.9%), either Medicare (53.3%) or Medicaid (10.6%). Most patients were discharged from hospital within 7 days (70.5%) and under routine circumstances (63.6%). Hospitals where inpatient care was provided were frequently large (62.3%), private non-profit (71.1%), and metropolitan teaching hospitals (72.1%).

**Table 1:** Demographics of SPS index hospitalizations, 2014

Characteristic	Index Events *n* (%) *n* = 703
Age	
< 40	111 (15.8)
40–49	138 (19.6)
50–59	186 (26.5)
60+	268 (38.1)
Sex	
Male	254 (36.1)
Female	450 (63.9)
Primary payer^a^	
Private insurance	232 (32.9)
Medicare	375 (53.3)
Medicaid	75 (10.6)
Median household income	
$66,000+	152 (21.6)
$51,000 - $65,999	154 (21.9)
$40,000 - $50,999	192 (27.4)
$1 - $39,999	200 (28.5)
Length of stay	
0–7 days	495 (70.5)
> 7 days	208 (29.5)
Discharge disposition^a^	
Routine	447 (63.6)
Transfer: short-term hospital	**
Transfer: other type of facility	106 (15.0)
Home health care	132 (18.8)
Comorbidities	
0–2	287 (40.8)
3–4	247 (35.1)
5+	170 (24.1)
Bed size of hospital	
Small	89 (12.6)
Medium	176 (25.0)
Large	438 (62.3)
Control/ownership of hospital^a^	
Private, not-for-profit	500 (71.1)
Private, investor-owned	96 (13.7)
Teaching status of hospital^a^	
Metropolitan teaching	507 (72.1)
Metropolitan non-teaching	150 (21.3)

^a^Some categories excluded due to small sample size

**10 or fewer observations – data suppressed

Based on HCUP single-level Clinical Classifications Software [11], “other hereditary and degenerative nervous system conditions” was the most frequently recorded group of primary reasons for index SPS hospitalization (29.5%), with nearly all (94.2%) admissions in this group attributable to SPS (Table 2). Other leading primary reasons for index hospitalization included ‘diabetes complications’ (5.1%), ‘septicemia’ (3.9%), ‘other nervous system disorders’ (3.7%), and ‘spondylosis, intervertebral disc disorders, and other back problems’ (2.4%).

**Table 2:** The 10 most common primary reasons for SPS index hospitalization

All Index Encounters Reason for inpatient admission (*n* = 703)	*n* (%)
Other hereditary and degenerative nervous system conditions	207 (29.5)
Diabetes mellitus with complications	36 (5.1)
Septicemia	27 (3.9)
Other nervous system disorders	26 (3.7)
Spondylosis; intervertebral disc disorders; other back problems	17 (2.4)
Complication of device; implant or graft	16 (2.3)
Meningitis	15 (2.1)
Respiratory failure; insufficiency; arrest	12 (1.7)
Other gastrointestinal disorders	12 (1.7)
Epilepsy; convulsions	11 (1.6)

### Readmissions within 30 Days

There were 66 (9.4%) patients hospitalized with SPS who were readmitted for any cause within 30-days of index encounter discharge: 5.3% of them were readmitted to the same hospital and 4.1% were readmitted to a different hospital (Table 3). Readmissions were predominantly for ‘diabetes with complications’ (24.2%), SPS and ‘other hereditary and degenerative nervous system conditions’ (% suppressed), ‘unclassified events’ (% suppressed), and ‘complication of devices; implants or grafts’ (% suppressed). Median time to the first readmission within 30 days was 10.0 days (interquartile range (IQR): 5.4–20.9). The median time to first readmission to the same hospital was 12.2 days (IQR: 3.2–22.5), whereas it was 7.7 days (IQR: 7.1–11.9) for readmissions to different hospitals. Nearly all readmissions attributed to diabetes complications (87.5%) were to different hospitals than where index inpatient care was received. Diabetes complications accounted for approximately half (49.6%) of all readmissions to different hospitals. All patients in our sample readmitted for SPS were readmitted to the same hospital from which they were previously discharged.

**Table 3:** The 10 most common primary reasons for readmission within 30 days of discharge – total and by readmission hospital

All Index Encounters Reason for Readmission (*n* = 66 / 703; Readmission Rate = 9.4%)	n (%)
Diabetes mellitus with complications	16 (24.2)
Other hereditary and degenerative nervous system conditions	**
Other nervous system disorders	**
Residual codes; unclassified	**
Complication of device; implant or graft	**
Other nutritional; endocrine; and metabolic disorders	**
Epilepsy; convulsions	**
Septicemia	**
Mood disorders	**
Fluid and electrolyte disorders	**
Index Encounters Readmitted to Same Hospital Reason for Readmission (*n* = 37 / 703; Readmission Rate = 5.3%)	n (%)
Other hereditary and degenerative nervous system conditions	**
Residual codes; unclassified	**
Complication of device; implant or graft	**
Other nutritional; endocrine; and metabolic disorders	**
Septicemia	**
Fluid and electrolyte disorders	**
Pneumonia	**
Other and unspecified benign neoplasm	**
Other connective tissue disease	**
Other fractures	**
Index Encounters Readmitted to Different Hospital++ Reason for Readmission (*n* = 29 / 703; Readmission Rate = 4.1%)	n (%)
Diabetes mellitus with complications	14 (49.6)
Epilepsy; convulsions	**
Peri-; endo-; and myocarditis; cardiomyopathy	**
Other nervous system disorders	**
Sickle cell anemia	**
Skin and subcutaneous tissue infections	**
Mood disorders	**

**10 or fewer observations – data suppressed

++Only 7 primary reasons for hospitalization within strata

### Factors associated with hospital readmission

Relative to males, females had an increased odds of being readmitted within 30 days of inpatient discharge (odds ratio (OR), 3.29; 95% confidence interval (CI): 1.22–8.87) (Table 4). Compared to all other discharge types, patients discharged to home under routine circumstances were significantly less likely to be acutely readmitted to any (OR, 0.26; CI: 0.10–0.64) or different hospitals (OR, 0.18; CI: 0.04–0.89). Patients who received care at private, investor-owned hospitals were significantly more likely (OR, 10.87; CI: 2.03–58.25) to be acutely readmitted to a different hospital than those receiving care at other hospitals, including non-profit care facilities (OR, 0.26; CI: 0.05–1.41). No other demographic, clinical, or hospital characteristic was associated with all-cause readmission within 30 days of discharge.

**Table 4:** Odds of readmission according to demographic, clinical, and care setting characteristics

Characteristic	Any Readmission		Readmission to Different Hospital	
	*p*-value^b^	OR	*p*-value^b^	OR
Age				
< 40	0.3284	Reference	0.3284	Reference
40–49		0.31 (0.09-1.12)		0.59 (0.08–4.53)
50–59		0.52 (0.18-1.48)		0.64 (0.10–3.99)
60+		0.69 (0.21-2.34)		1.51 (0.19–12.36)
Sex				
Male	0.0231	Reference	0.0231	Reference
Female		3.29 (1.22–8.87)*		2.57 (0.52–12.65)
Primary payer^a^				
Private insurance	0.0773	0.41 (0.15–1.13)	0.0773	0.29 (0.05–1.70)
Medicare	0.2300	1.81 (0.74–4.42)	0.2300	1.88 (0.38–9.23)
Medicaid	0.6354	1.33 (0.44–3.98)	0.6354	2.13 (0.41–11.18)
Median household income^a^				
$66,000+	0.5110	Reference	0.5110	Reference
$51,000 - $65,999		1.61 (0.48–5.33)		2.10 (0.18–24.30)
$40,000 - $50,999		0.95 (0.28–3.24)		0.89 (0.05–14.82)
$1 - $39,999		2.54 (0.66–9.77)		8.87 (0.81–96.73)
Length of stay				
0–7 days	0.3016	Reference	0.3016	Reference
> 7 days		1.59 (0.65–3.90)		0.88 (0.18–4.28)
Discharge disposition^a^				
Routine	0.0244	0.26 (0.10–0.64)**	0.0244	0.18 (0.04–0.89)*
Transfer: short-term hospital	0.4935	3.28 (0.34–31.36)	0.4935	8.38 (0.73–95.73)
Transfer: other type of facility	0.0848	2.40 (0.96–5.97)	0.0848	1.14 (0.23–5.74)
Home health care	0.2774	2.63 (0.75–9.18)	0.2774	4.66 (0.71–30.70)
Comorbidities				
0–2	0.6717	Reference	0.6717	Reference
3–4		0.81 (0.34–1.97)		0.65 (0.15–2.83)
5+		1.64 (0.46–5.83)		2.90 (0.43–19.70)
Bed size of hospital				
Small	0.4433	Reference	0.4433	Reference
Medium		3.28 (0.57–18.76)		4.06 (0.33–50.36)
Large		1.62 (0.37–7.13)		0.94 (0.11–8.05)
Control/ownership of hospitala				
Private, not-for-profit	0.8673	0.89 (0.24–3.33)	0.8673	0.26 (0.05–1.41)
Private, investor-owned	0.4248	2.44 (0.53–11.25)	0.4248	10.87 (2.03–58.25)**
Teaching status of hospitala				
Metropolitan teaching	0.5039	1.38 (0.53–3.57)	0.5039	1.75 (0.35–8.77)
Metropolitan non-teaching	0.8803	1.08 (0.42–2.79)	0.8803	0.83 (0.17–4.17)

Abbreviation: *OR* odds ratio

^a^Some categories excluded due to small sample size. ^b^Chi-square test

**p < 0.01, *p < 0.05

## Discussion

Stiff person syndrome is a rare and progressive neurological disorder that if left untreated may contribute to significant patient disability and poor quality of life. Considerable advancements have been made in understanding SPS disease etiology, with strong evidence demonstrating that GAD65 antibodies may serve as an excellent diagnostic indicator and that patients with SPS are also often diagnosed with autoimmune disorders such as diabetes, hypothyroidism, and pernicious anemia [6, 12]. Despite the pathophysiology of classic SPS not being fully understood, research has shown that the disorder may be effectively treated using pharmacotherapies, ranging from antispasticity and γ-aminobutyric acid enhancing medications to the use of immunotherapies [4]. It is widely believed that SPS is underdiagnosed [4, 13]; however, much of the current knowledge about SPS epidemiology and healthcare utilization originate from case studies and small observational studies, making it difficult to precisely estimate disease burden and health system impacts. Using NRD data, we identified more than 700 individuals who were admitted to hospital with SPS and discharged alive between January and November 2014 for the purposes of characterizing their inpatient care and readmissions within 30 days of hospital discharge. Our primary findings were that individuals with SPS are often admitted to hospital as a result of SPS and diabetes complications, and that acute readmissions among individuals with SPS are relatively common. Secondary findings include: (1) readmissions within 30 days were largely due to diabetes complications, (2) nearly all readmissions attributed to diabetes complications were to different hospitals, (3) female sex and receipt of care at private, investor-owned hospitals was associated with increased odds of being acutely readmitted and acutely readmitted to a different hospital, respectively, and (4) being discharged under routine circumstances (such as to home or self-care) was associated with decreased odds of being readmitted within 30 days. To our knowledge, this is the largest nationally representative study of SPS, which provides timely and much needed data on the epidemiology and inpatient care for this orphan disorder.

Published studies regularly report the prevalence of SPS to range from 0.5 to 2 cases per million habitants [3, 8, 14, 15]; however, to date, few population-based studies have actually been conducted to estimate the true burden of SPS [8, 13, 14]. Between 2000 and 2015, the British Neurological Surveillance Unit identified 119 individuals with SPS from the United Kingdom, suggesting a disease prevalence of 1–2 cases per million people [8]. More recently, the first reported epidemiological study of SPS in Sub-Saharan Africa estimated SPS prevalence to be 0.9 cases per 1,000,000 individuals living in the Kilimanjaro region [14]. These prevalence estimates are conservative compared to the 20 cases that were identified from a population of 2 to 3 million in the areas surrounding Heidelberg, Germany over a period of 10 years, which highlight population risk differences and the likely underdiagnosis of SPS [13]. Our finding that 703 individuals with SPS were hospitalized between January and November 2014 in the United States suggests that the true prevalence of SPS in North America may exceed 2 cases per million habitants, which coincides with expert opinion that SPS may not be as rare as previously thought [4]. There are conflicting reports regarding SPS prevalence by sex, with some studies reporting that the disorder equally affects men and women [16] and others finding that SPS disproportionally affects women [6, 17] and men [14]. Our predominantly female (63.9%) cohort supports prior reports that nearly two thirds of SPS patients are women [6, 17]. However, it is important to acknowledge that observed sex differences in SPS prevalence may reflect underlying differences in population disease risk or health behaviors, and that additional studies are required to characterize populations at greatest risk of SPS.

Our finding that SPS and diabetes complications were the most common reasons for index hospitalization reaffirms the association and autoimmune link between SPS and type 1 diabetes (30–50% of all SPS patients are reported to have type 1 diabetes and the majority of SPS patients have elevated titer antibodies against GAD) [8, 18–20]. It is possible that acute readmissions in SPS result from planned specialty care, such as admission for intravenous immunoglobulin or plasmapheresis immunotherapies at academic medical centers, or gaps in discharge planning. However, our findings that diabetes complications were the largest driver of re-hospitalization within 30 days of inpatient discharge and that such readmissions were almost always to different hospitals suggest that these SPS readmissions resulted from medical emergencies, possibly diabetic ketoacidosis. Such emergencies have been widely reported among patients with both SPS and diabetes, and would cause individuals to seek medical care at their closest hospital [21–23].

Patients with SPS and diabetes are at risk of diabetic ketoacidosis, a preventable life-threatening condition that often leads to hospitalization [24, 25]. This raises the important question about whether a proportion of readmissions in SPS are avoidable. A recent 5-year retrospective study of 367 patients at a United States tertiary academic medical center identified history of depression or substance/alcohol abuse, and self-pay/publicly funded insurance as significant independent predictors of readmission for diabetic ketoacidosis [24]. Authors propose that readmissions for diabetes complications may be avoided by providing target interventions, including tighter glycemic control, to patients classified as high risk for recurrent diabetic ketoacidosis according to an objective scoring systems based on established risk factors [24]. Implementation of such interventions when treating inpatients with SPS and comorbid diabetes may directly translate into significant cost savings for healthcare systems, including a reduction in readmission penalties for neurology services where SPS are routinely admitted, as well as improved health outcomes and quality of life for patients.

Female sex (*p* > 0.01) and receipt of care for SPS at private, investor-owned hospitals (*p* < 0.01) were the only independent predictors that we found to be positively associated with readmission within 30 days of discharge. Small samples sizes in compared groups contributed to uncertainty around parameter estimates for examined associations, which precludes making any assertions regarding the association of these factors with readmissions in SPS. Nevertheless, these findings provide useful benchmark data for future studies that examine whether readmissions in SPS are preventable. Relative to other discharge dispositions, we found that individuals discharged under routine circumstances had decreased odds of being acutely readmitted within 30 days, including to different hospitals. This was likely attributable to this subpopulation having fewer comorbid conditions and being younger than those discharged to other facilities and with increased healthcare needs.

There are numerous strengths to our study. We used a large, nationally representative dataset of inpatient care that included longitudinal follow-up data on readmissions occurring within the same calendar year. Using these data and survey weighting methods, we were able to precisely estimate and describe annual inpatient care for SPS from a population of more than 300 million individuals. In-depth data on demographic, clinical, and hospital characteristics allowed us to describe the population with SPS that is most commonly admitted to hospital, including their primary causes of hospitalization and re-hospitalization, and factors associated with acute readmission. Inpatient care was most often received at large (62.3%) metropolitan teaching (72.1%) hospitals that are neurologist-rich and presumed leaders in adhering to best clinical practices and in leveraging advances in knowledge to improve disease diagnosis and treatment efficiencies. As such, it is likely that suspected SPS diagnoses made at these hospitals were confirmed using validated SPS diagnostic criteria and serum anti-GAD antibody testing [4, 26].

Notwithstanding our study’s strengths, certain limitations should be considered when interpreting our findings. Small sample sizes precluded the reporting of most rates of primary cause of readmission and hindered us from completing adequate multivariable modeling and controlling for potential sources of confounding in our crude estimates. Study data did not permit longitudinal follow-up for individuals admitted or transferred to out-of-state hospitals, which likely resulted in our underestimation of the true number of index SPS hospitalizations. Despite these limitations, our study highlights the relatively large number of individuals, mainly women, living in the United States who are hospitalized in a given year with SPS, and is the largest epidemiological study to characterize inpatient care and health service utilization by this population. Despite many inpatients receiving care at large, metropolitan teaching hospitals, laboratory anti-GAD antibody testing and prior medical history data were unavailable and therefore could not be used in our selection of index SPS cases, leading to possible misclassification of SPS diagnoses. Since the rarity of SPS may lead to the disorder being over- or under-diagnosed in clinical practice, it is possible that a portion of hospitalized individuals identified as having SPS in our study did not actually have SPS and that some true cases of SPS were omitted from our study cohort. Taken together, study limitations preclude us from definitively knowing the true number of SPS cases included in our study cohort or the number of cases that went undetected. Nevertheless, the demographics of our study population are consistent with those reported by other studies of SPS in the United States [6, 20].

## Conclusions

In summary, using a large nationally representative readmission database from the United States, we found that readmissions in SPS are relatively common and may be attributed to complications of the disorder or associated comorbidities such as diabetes. Acute readmissions to different hospitals may result from unavoidable medical emergencies in the outpatient setting; however, may also result from planned specialty care or gaps in discharge planning. Study replication using other available health data is warranted; however, our preliminary estimates of disease burden suggest that the true prevalence of SPS may be higher than previously thought. Future studies that examine the extent to which readmissions in SPS may be prevented are required.

## Abbreviations

AHRQ: Agency for Healthcare Research and Quality
CI: confidence interval
GAD: glutamic acid decarboxylase
HCUP: Healthcare Cost and Utilization Project
ICD-9: International Classification of Diseases, Ninth Revision
IQR: interquartile range
NRD: Nationwide Readmissions Database
OR: odds ratio
SPS: Stiff person syndrome
